# Supplementary material for: The KDM6 histone H3K27me3 demethylase inhibitor GSK-J4 induces metal and stress responses in multiple myeloma cells
Source: RSC Chem Biol. 2026 Jul 10;7(10):2014–27. doi: 10.1039/d6cb00162a (PMC13470865; doi:10.1039/d6cb00162a)
Supplement: Supplementary file 1 [file CB-007-D6CB00162A-s001.pdf]

Supplementary Information (SI) for RSC Chemical Biology.
This journal is © The Royal Society of Chemistry 2026

Supplementary Figure 1: GSK-J4 and GSK-J5 dose-response curves in Multiple Myeloma cell lines.

Myeloma cell lines were cultured for 96 hours in the presence of different concentrations of GSK-J4 or GSK-J5 (n=3). Cell viability was measured using resazurin-based PrestoBlue assay (ThermoFisher) according to the manufacturer's instructions. Plates were read on a FLUOstar Optima (BMG) with excitation filter at 535nm and emission filter at 615nm. Dose-response curves are plotted as % cell viability over solvent control (0.1% v/v) DMSO in media.

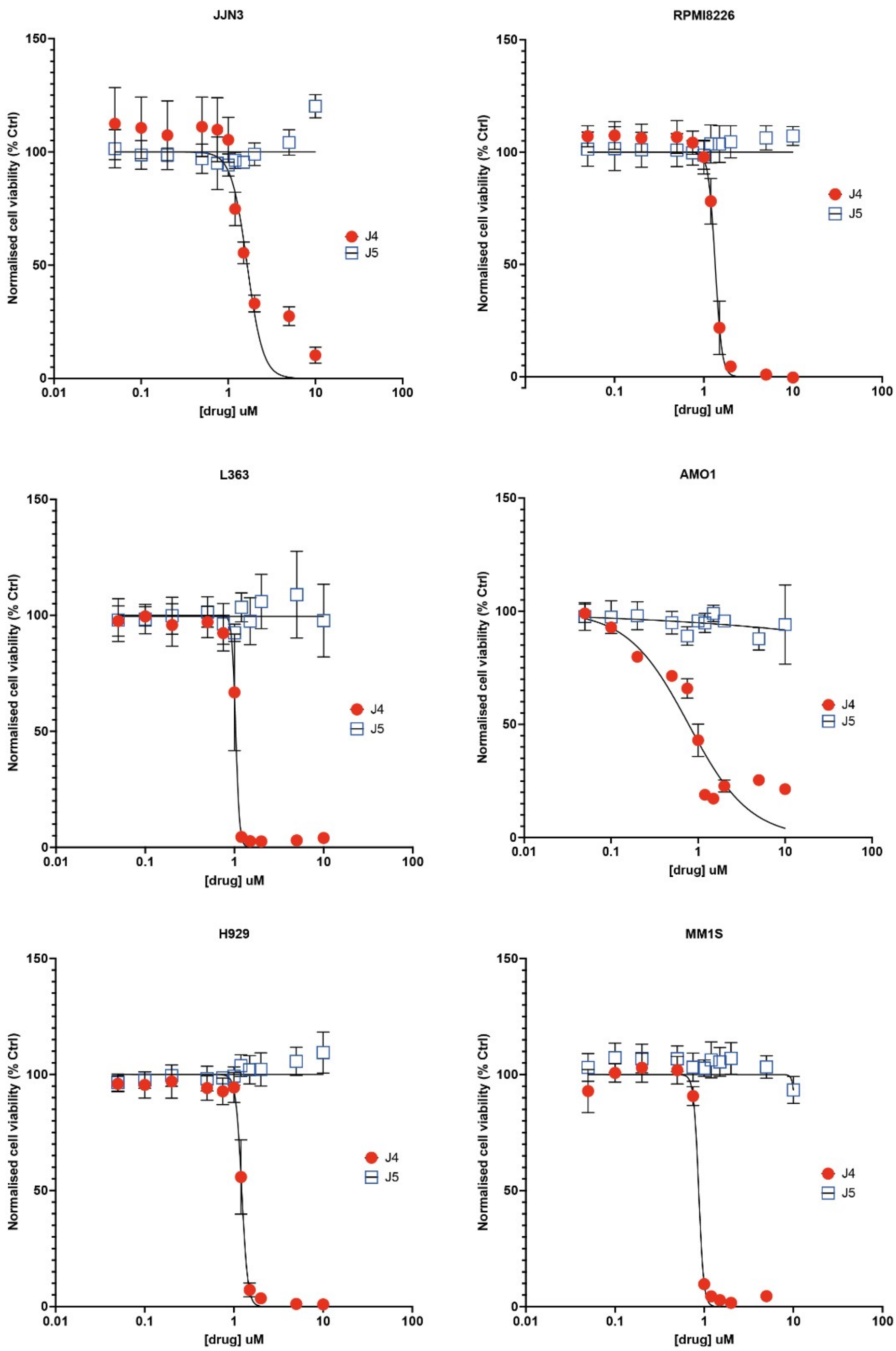

Supplementary Figure 2: Antiproliferative effects of GSK-J4 measured by EDU incorporation.

Six multiple myeloma cell lines were dosed with either 2µM GSK-J4, GSK-J5, or with 0.1% DMSO as a control for either 24 or 36 hours. Cell proliferation by DNA incorporation of EDU was measured using the Click-IT™ EDU assay (ThermoFisher) according to manufacturer's instructions and analysed using flow cytometry whereby EDU is identified as a positive FITC signal.

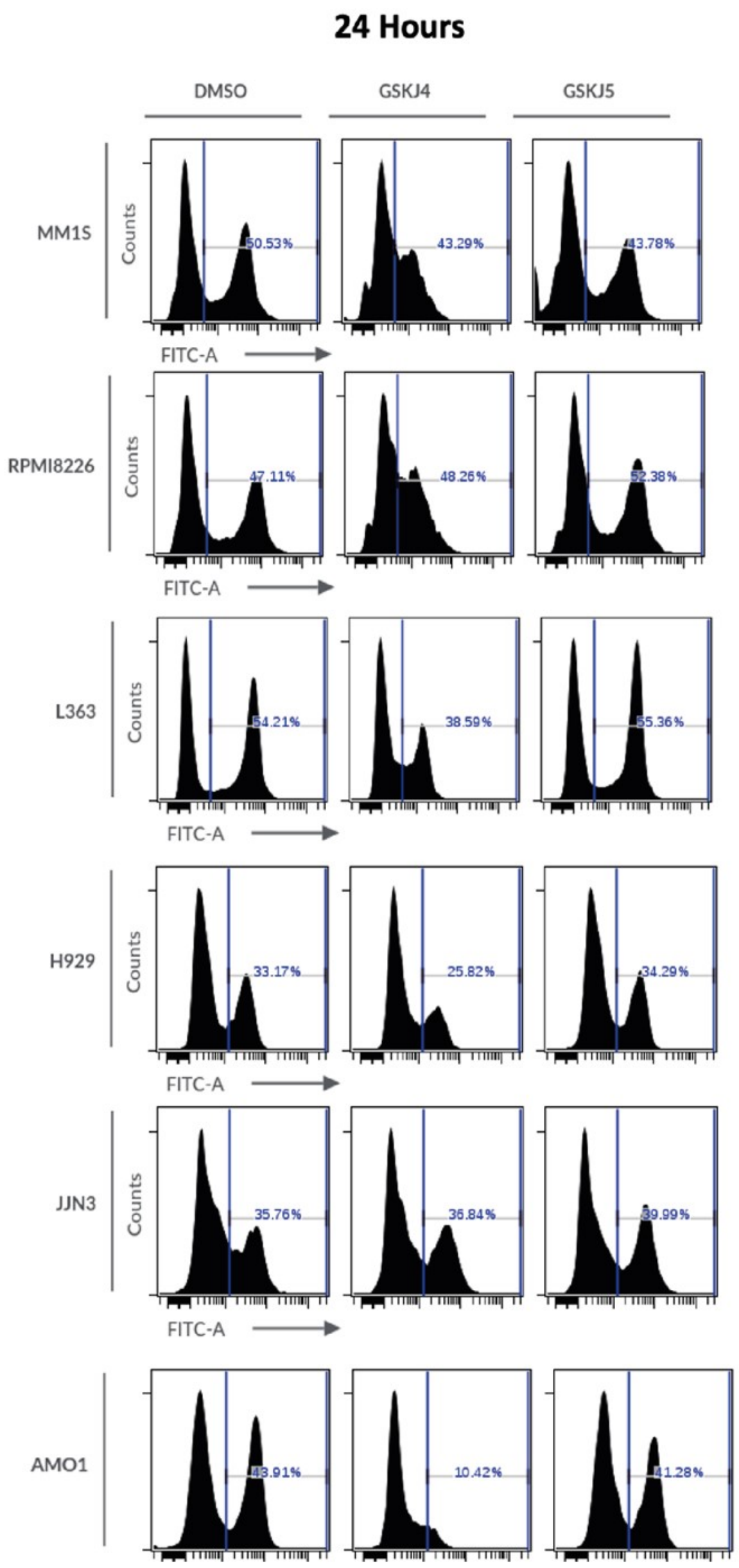

Supplementary Figure 3: Cell-death effects of GSK-J4 measured by Annexin V and PI staining.

Six multiple myeloma cell lines were dosed with either 2µM GSK-J4 or a 0.1% DMSO as a control for either 24 or 36 hours. Cells were stained with the eBioscience™ Annexin V Apoptosis Detection kit (Invitrogen) following manufactures instructions. Cell death was measured by Annexin V (FITC) and propidium iodide staining by flow cytometry with emission at 520nm and 580nm respectively. Each plot is representative of 10,000 events.

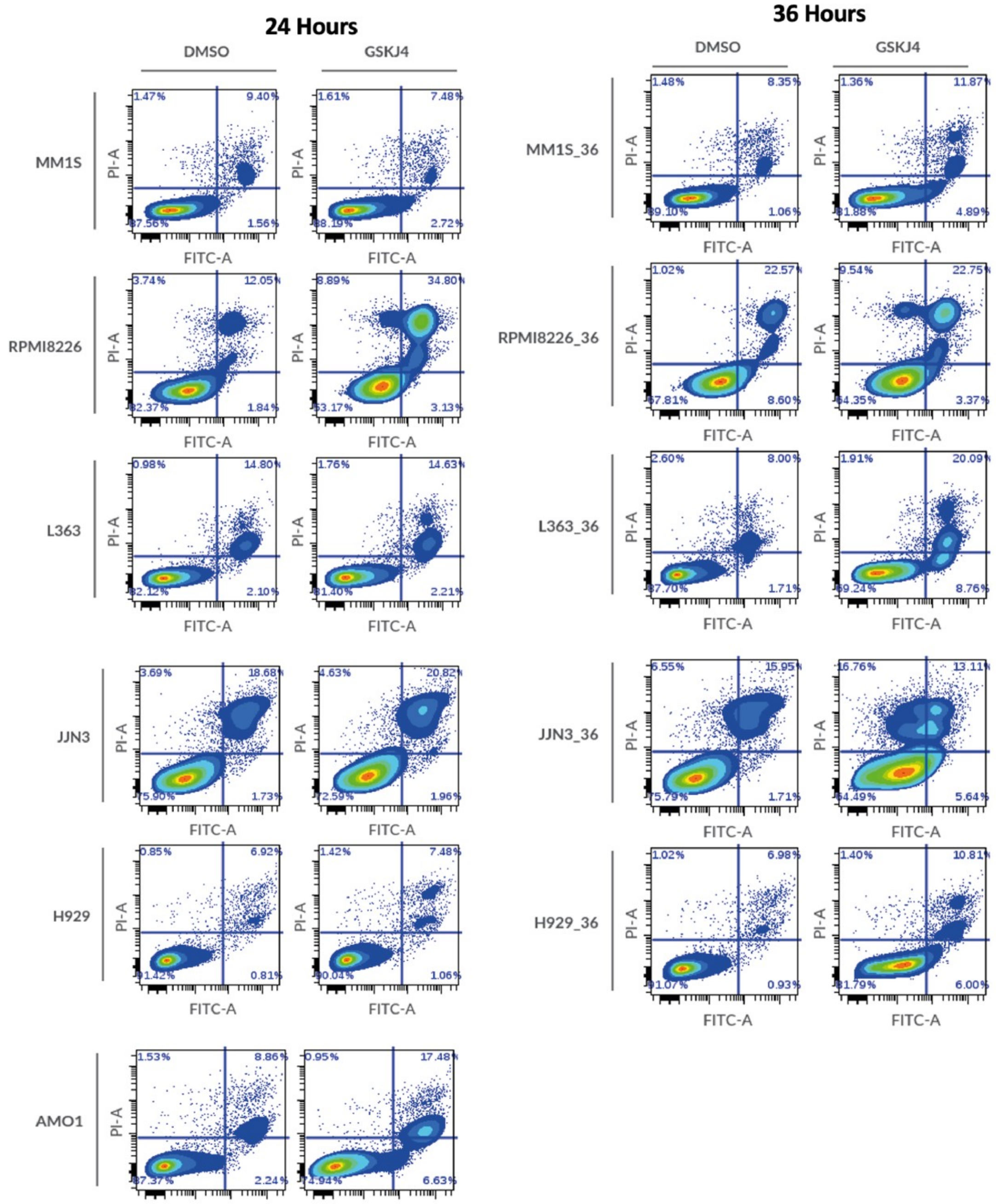

Supplementary Figure 4: Annexin V (FITC) and Propidium Iodide (PI) Staining of MM cells dosed with GSKJ4 for 72hrs

Four multiple myeloma cell lines were dosed with either 2µM GSK-J4, GSK-J5 or with 0.1% DMSO as a control for 72 hours. Cells were stained with the eBioscience™ Annexin V Apoptosis Detection kit (Invitrogen) following manufactures instructions. Cells death was measured by positive Annexin V (FITC) and propidium iodide (PI) staining by flow cytometry with emission at 520nm and 580nm respectively. Each plot is representative of 10,000 events.

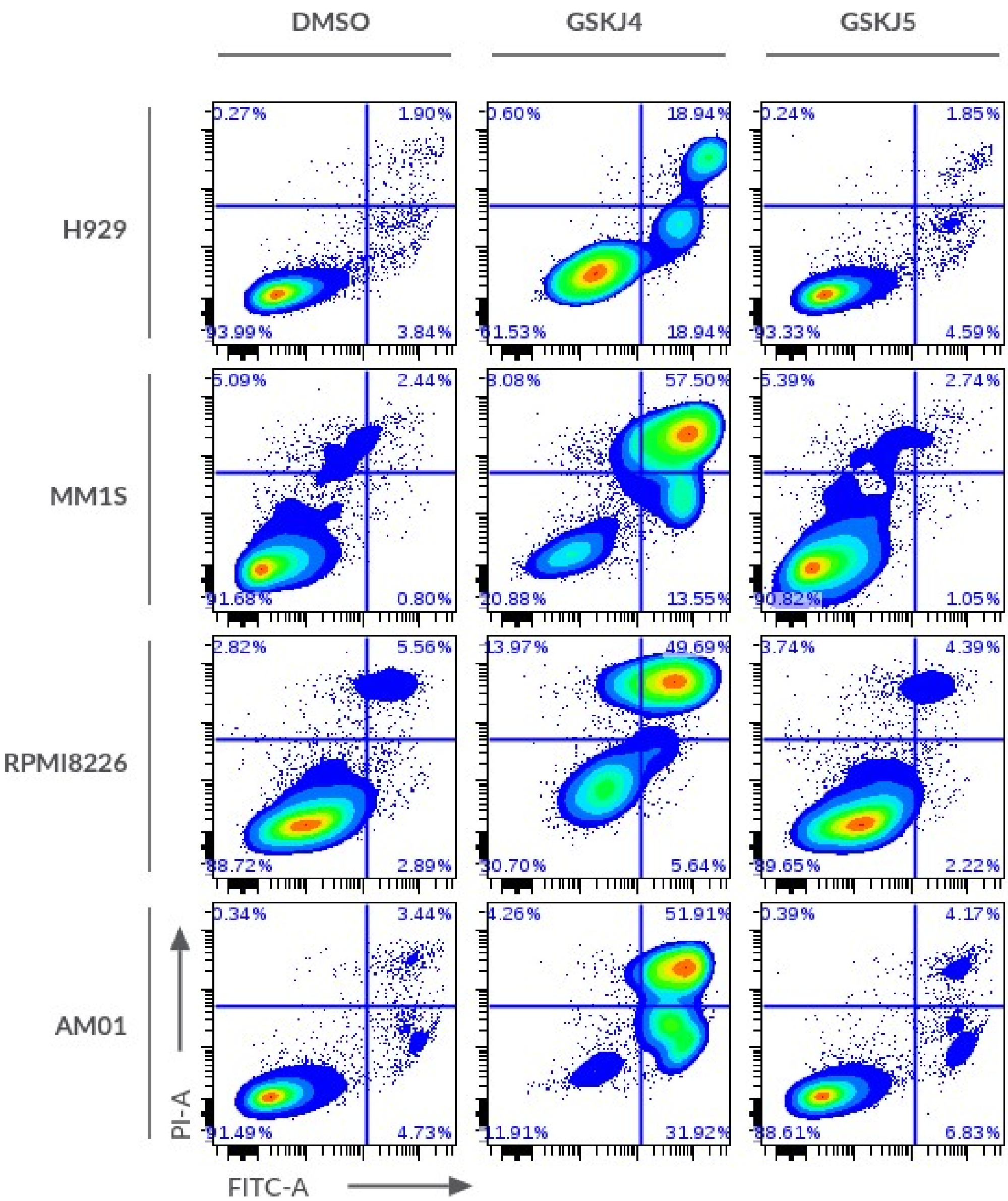

Supplementary Figure 5

Heatmap depicting metallothionein and SLC expression levels (RNAseq data) upon GSK-J4 treatment in different cellular systems. Publicly available data from NK cells, Th17 cells, Neurobloastoma cells and Adenocarcinoma cell lines can be accessed under the GEO database accession numbers GSE156423, GSE127767, GSE110709, GSE133970, respectively.

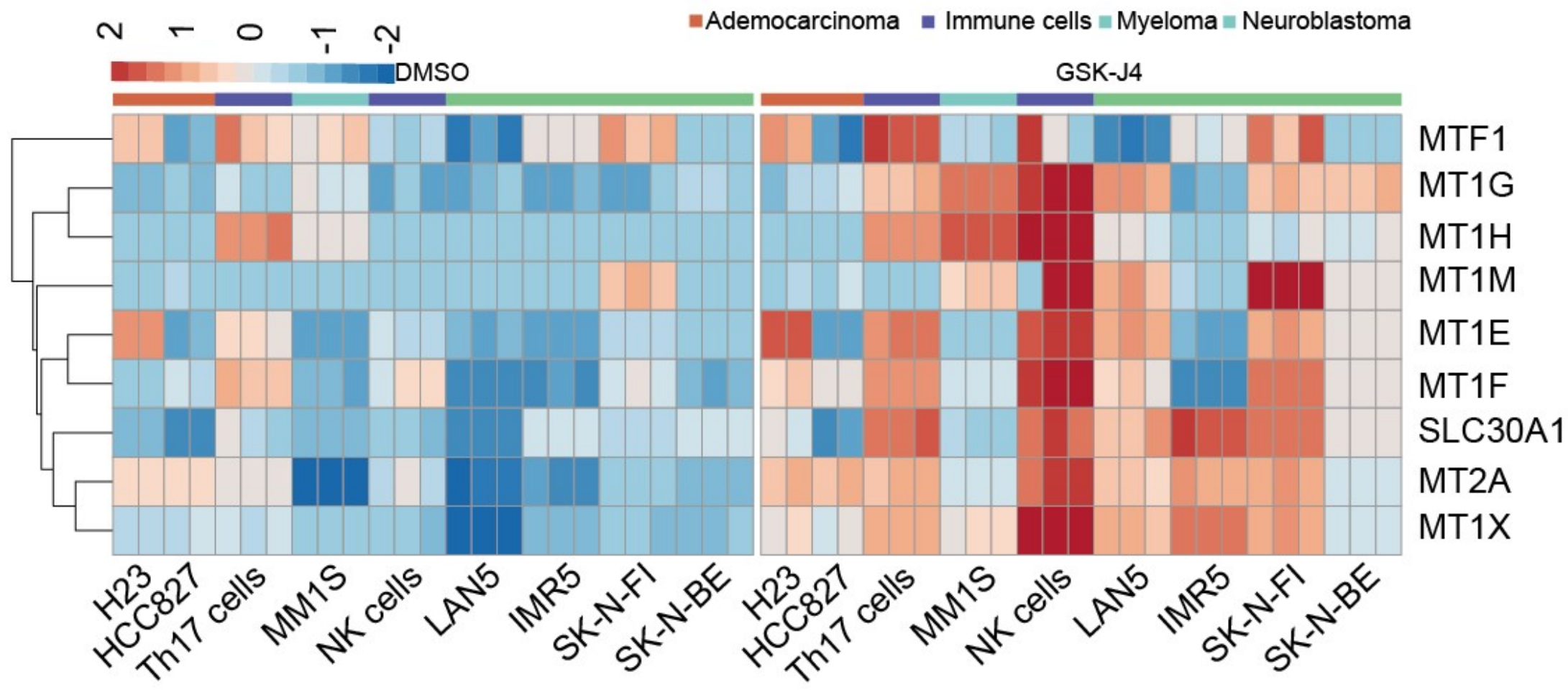

Supplementary Figure 6

UMAP showing the distribution of cell types in Myeloma patient bone marrow samples. UMAP plots showing the expression of MT1X (left panels) and MT1G (right panels) treated with either DMSO or GSK-J4 for 24 hours.

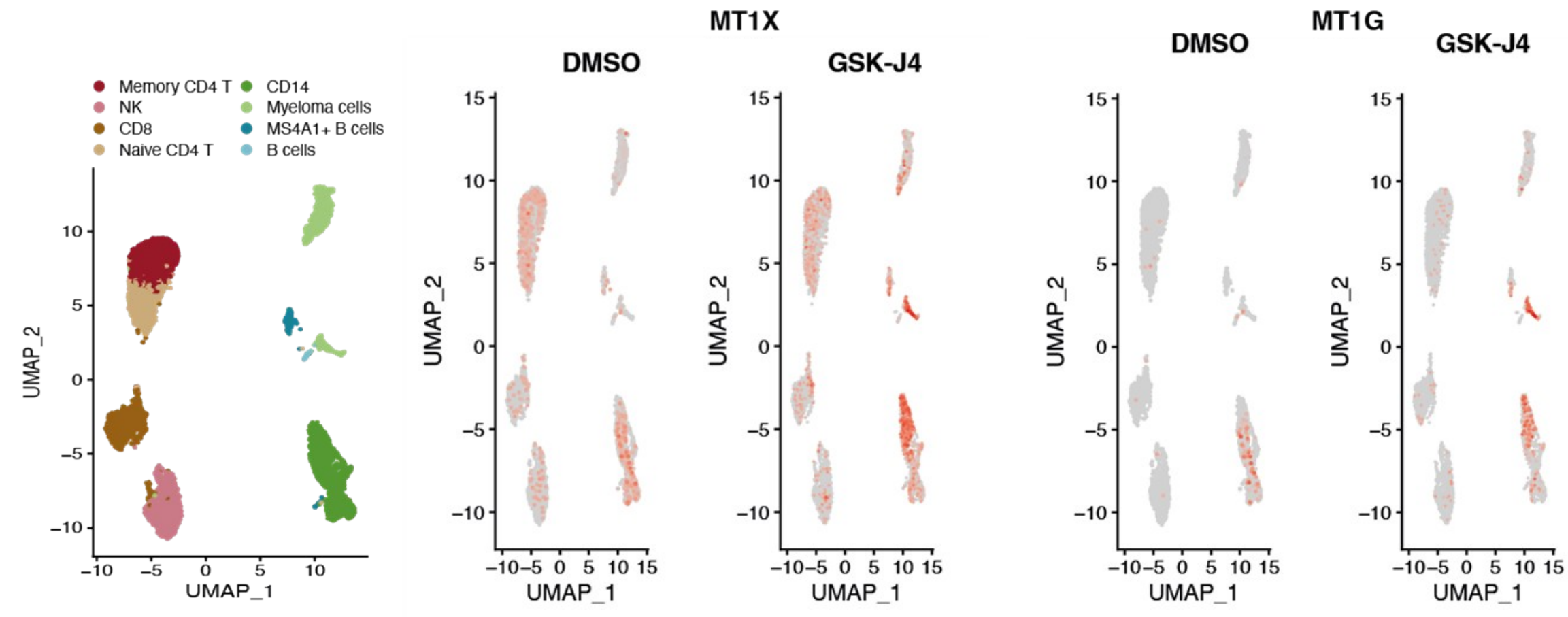

Supplementary Figure 7: Determination of Zn^{2+} binding to GSK-J4

A, B: To investigate the binding affinities of GSK-J4 and GSK-J5 towards Zn^{2+}, both compounds were prepared with 10, 1, or 0.1 equivalents of $ZnCl_2$ and their organometallic complex formation was assessed by MALDI-ToF mass spectrometry. Whereas GSK-J4 displayed concentration-dependent intensity of peaks corresponding to $[M+ZnCl]^+$adducts **(A)**, no Zn-bound species were detected for GSK-J5 **(B)** at any molar ratio of $ZnCl_2$. **C, D:** To quantify the observed difference in Zn^{2+} binding, ^1H NMR spectroscopic titrations were performed [11], wherein aliquots of $Zn(OTf)_2$ were titrated against solutions of GSK-J4 and GSK-J5 in a 1:1 mixture of d^3-MeCN:D_2O. For GSK-J4 the addition of increasing equivalents of Zn^{2+} induced significant chemical shift perturbations of signals in the aromatic region proximal to the proposed cation binding site **(C)**, whilst no shift was observed for GSK-J5 **(D)**. **E:** Bindfit analysis [22]of the GSK-J4 chemical shift perturbations determined a 1:1 GSK-J4:Zn^{2+} association constant in excess of 1500 M^{-1}. In contrast, analogous Zn^{2+}titration experiments with GSK-J5 elicited no perturbations in the chemical shift of any proton signal, suggesting negligible Zn^{2+} binding. Taken together, these results strongly suggest GSK-J4 binds Zn^{2+} far stronger than GSK-J5.

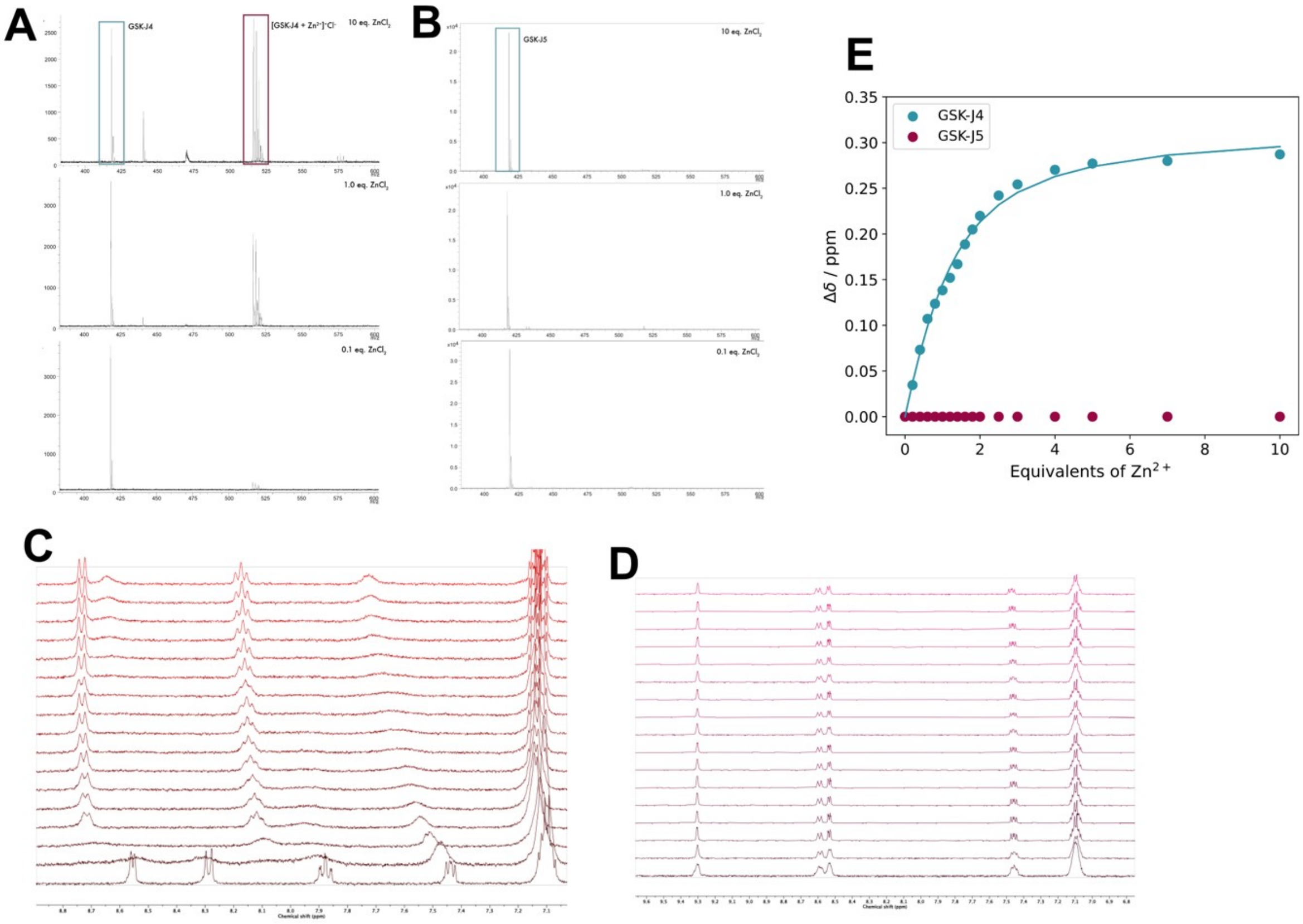

[1] A. Docker, H. Min Tay, *Chem. Eur. J.* 2024, 30, e202402844.
[2] Supramolecular: http://supramolecular.org
Hibbert2016: D. Brynn Hibbert and Pall Thordarson, Chem. Commun., 2016, 52, 12792-12805.
